# ManyFishes: a big team science collaboration on fish comparative cognition

**DOI:** 10.1007/s10071-025-02031-3

**Published:** 2025-12-16

**Authors:** Laurent Prétôt, Christian Agrillo, Benjamin C. Bluck, María J. Cabrera-Álvarez, Yseult Héjja-Brichard, Kyndal Irwin, Annabell Klinke, Tyrone Lucon-Xiccato, Cait Newport, Ronen Segev, Justin Yeager

**Affiliations:** 1 Department of Psychology and Counseling, Pittsburg State University, Pittsburg, KS 66762 USA; 2 Department of General Psychology, University of Padova, Padova, Italy; 3 Padova Neuroscience Center, Padova, Italy; 4 International Centre for Ecohydraulics Research, Faculty of Engineering and Physical Sciences, University of Southampton, Southampton, UK; 5 School of Natural Sciences, Macquarie University, Sydney, Australia; 6 FishEthoGroup Association, Faro, Portugal; 7 Centre of Marine Science (CCMAR/CIMAR LA), Faro, Portugal; 8 Department of Interdisciplinary Life Sciences, University of Veterinary Medicine Vienna, Vienna, Austria; 9 Department of Behavioural and Cognitive Biology, University of Vienna, Vienna, Austria; 10 Department of Biology, Texas State University, San Marcos, USA; 11 Department of Life Sciences and Biotechnology, University of Ferrara, Ferrara, Italy; 12 Department of Biology, University of Oxford, Oxford, UK; 13 Department of Life Sciences, Ben Gurion University of the Negev, Beer Sheva, Israel; 14 Grupo de Investigación en Biodiversidad, Medio Ambiente y Salud (BIOMAS), Facultad de Ingenierías y Ciencias Aplicadas, Universidad de Las Américas, Quito, Ecuador

**Keywords:** Big team science, Comparative cognition, Fish cognition, Fish diversity, Fishes, Metascience

## Abstract

Fishes are among the oldest and most diverse groups of vertebrates, encompassing a vast array of species that differ in morphology, ecology, and behavior. While such diversity can pose challenges to comparative cognition research, it can also offer valuable insights into how different ecological and evolutionary pressures shape cognitive abilities. As the earliest diverging extant vertebrates, and the simplest neural architecture, fishes also provide a critical window into the origins and evolution of vertebrate intelligence. However, despite their potential, we have only just begun to scratch the surface of what fish cognition can reveal, in part due to practical limitations that have constrained cross-species comparisons, including the use of small, single-species samples and a lack of standardized testing procedures. To address these challenges, we introduce *ManyFishes*, the first big team science collaboration dedicated to comparative cognition and behavior in fishes. Here, we discuss the benefits, potential, challenges and solutions, and impact of this large-scale collaborative effort. Like other big team science initiatives, ManyFishes relies on the ability to establish an infrastructure that facilitates communication and coordination among collaborators from diverse backgrounds, while promoting openness, transparency, and reproducibility in fish cognition research. With this paper, we aim to raise awareness of the ManyFishes initiative and invite researchers to join, contribute to, and benefit from this large-scale collaborative effort in future projects.

## Importance of big team science for comparative cognition

Large-scale collaborations known as big team science enable the investigation of complex, fundamental research questions that are beyond the reach of individual laboratories by uniting researchers from varied disciplines, career stages and educational backgrounds and pooling their resources and expertise. Comparative cognition, in particular, has benefited from the big team science movement, with established networks representing a wide range of animal groups, including ManyBabies (developmental psychology; Frank et al. 2017; Lucca et al. 2025), ManyBirds (avian cognition and behavior; Lambert et al. 2022), ManyDogs (canine science; ManyDogs Project et al. 2023a), ManyGoats (goat behavior and cognition; link: https://www.themanygoatsproject.com/), ManyManys (cross-taxon comparative cognition; Alessandroni et al. 2024a, b), ManyPrimates (primate cognition; Many Primates et al. 2019), ManyZoos (zoo management across taxa; Barrett et al. 2025) and, more recently, ManyOtters (otter cognition research; link: https://manyotters.github.io/). Although these networks differ in the specifics (e.g., animal models, theories, research questions, methods), they all pursue a common goal: using larger and more diverse samples to enhance the reproducibility, replicability, and robustness of research findings in their area of specialization. Such an approach is particularly crucial in cognitive research, where high levels of individual and species variability demand large, repeated, and diverse datasets to produce robust and broadly applicable insights.

Despite the clear potential benefits of big team science to comparative cognition research, *implementing* it can be very difficult (Alessandroni et al. 2024a, b). Such challenges include ensuring fair comparisons between species, dealing with variation across testing sites, and reconciling discrepancies that originate from the diverse theoretical and disciplinary backgrounds of the researchers involved. In addition, big team science projects can be extremely time-consuming, difficult to secure funding for, and not always perceived as valuable for career advancement. Due to the inherently highly collaborative nature of big team science networks, however, many of these issues can be equitably resolved thanks to thoughtful discussions, which leverage the diverse backgrounds and experiences of members. The future of big team science in the field of comparative cognition eventually lies in the success of different networks to identify the challenges that are specific to their thematic area and focal group and, more importantly, propose viable solutions to mitigate them.

An area of research that is currently experiencing significant growth, but suffers from comparability, reproducibility, and replicability problems, is the field of fish cognition. Fishes are of ancestral relevance to vertebrate phylogeny, with the common ancestor of tetrapods being a bony fish, and have undergone significant independent evolution and radiation since the split from tetrapods (Bshary and Brown 2014). Fishes are also an extremely diverse group, including a wide range of species that vary in morphology, habitat, diet, and behavior. In terms of cognitive abilities, fishes can learn novel information extremely quickly (often after a single trial; Blank et al. 2009; Lucon-Xiccato and Dadda 2014), and they can remember it for months, if not even years (Beukema 1970; Brown 2001). They can perform complex discrimination tasks based on multiple sensory modalities (Mackintosh and Sutherland 1963; Mitchell et al. 2015; Newport et al. 2016; Schluessel et al. 2012). Some species show the ability to solve complex tasks (Lucon-Xiccato et al. 2020; Mair et al. 2021), use tools (Brown 2012), and rely on mathematical principles to solve problems under uncertainty (Newport et al. 2024). Most social fishes can accurately judge the number of social companions in a group (Agrillo and Dadda 2007), recognize individual shoal mates (Kohda et al. 2015), and cooperate or deceive others (Milinski 1987; Plath et al. 2008). Studies examining some of the very core cognitive functions described for mammalian species, such as attention and inhibitory control, suggest similarities with fishes both at the performance and mechanism levels (Echevarria et al. 2011; Lucon-Xiccato 2024). There is even evidence that some fish species can outperform non-human primates in some dichotomous choice tasks (Salwiczek et al. 2012). Yet, differences from primates have also been observed. For example, there is still no evidence that fishes can learn abstract relational concepts (Newport 2021; Newport et al. 2015), a skill that is fundamental to human cognition. Also, interspecific differences have been reported among fishes tested in different cognitive tasks (e.g., Agrillo and Pecunioso 2024; Agrillo et al. 2012; Bisazza et al. 2014). Determining whether the cognitive capacities reported in certain fish species represent broader patterns across the group, reflect shared ancestry with other vertebrates, or constitute independent instances of convergent evolution, remains a complex and unresolved question. Given the extensive diversity, ancient history, and non-monophyletic nature of fishes, broad generalizations about “fish cognition” should thus be approached with caution.

Despite the increased number of studies (and discoveries) on fish cognitive capacities in recent decades, several experimental limitations continue to hinder our full understanding of their cognitive potential; those include the use of small single-species samples and a lack of standardized testing procedures (Salena et al. 2021), which can make cross-species comparisons challenging. For example, the sheer differences in body size – from proverbial minnows to sharks – contribute to difficulties in adapting even simple tasks across species, a problem not typically found in animal groups covered by other big team science networks. Furthermore, cognitive tests are traditionally designed for two-dimensional planes, which might be more suitable for working with terrestrial vertebrates than with aquatic organisms that often move freely within three-dimensional space. Fish cognition research thus faces the unique challenge of developing and adapting experiments and methodologies to account for fishes’ complex multi-dimensional environments (see Cohen et al. 2023; Gatto et al. 2017). As a result, testing methods may bias comparisons to other taxonomic groups, potentially leading to an underestimation of the full extent of fish cognitive abilities.

In this paper, the authors – representing *ManyFishes*, a recently formed big team science collaboration on comparative cognition and behavior in fishes (link: https://themanyfishes.github.io/) – argue that many of the challenges encountered in the field can indeed be successfully mitigated leveraging a big team science-style approach. We begin by reviewing the literature on fish cognition and identifying existing empirical and theoretical gaps that would benefit from big team science collaborations. Next, we introduce the ManyFishes consortium, providing an overview of its first empirical study – ManyFishes 1 – and outlining potential directions for future research. Finally, we discuss the challenges and solutions associated with this unique collaborative effort and consider its broader impact on other fields of fish science. Through this paper, we aim to inform the field about the ManyFishes initiative and encourage scholars to contribute to and benefit from this broad collaborative endeavor in their future projects.

## Fish cognition needs big team science

### Leveraging fish diversity to explore the evolution of cognition

Fishes are among the most diverse groups of vertebrates on the planet, comprising approximately 32,000 to 34,000 species and accounting for nearly 50% of all extant vertebrates (Burton and Burton 2017; Helfman et al. 2009). They have successfully adapted to inhabit virtually *all* aquatic environments, including oceans, seas, freshwater systems, temporary habitats, and even extreme environments such as hypersaline lakes, polar waters, and caves. Their remarkable diversity in morphology, ecology, and habitats makes them an ideal group to explore how such factors can shape cognitive abilities. Extant fishes are broadly divided into three main lineages – jawless, cartilaginous and bony fishes (including ray-finned and lobe-finned fishes) – with a relatively long separate evolutionary history. For example, the oldest and the most recent of these lineages are estimated to have diverged approximately 400 million years ago (Botella et al. 2007; Brazeau and Friedman 2015; Zhu et al. 2009). Following this extensive evolutionary history, fishes exhibit an impressive range of morphological variation. As an example, some fish species measure less than one centimeter in length (Kottelat et al. 2006), whereas others exceed 15 meters (see Rowat and Brooks 2012). Intriguingly, the variability of fishes is often visible within relatively small phylogenetic lineages. For example, within poeciliid fishes, the placenta, which determines a shift from pre- to post-fertilization provisioning, has evolved independently at least nine times (Furness et al. 2019, 2021; Pollux et al. 2009). Similarly, in Lamprologini cichlids, cooperative breeding has evolved five times (Dey et al. 2017). A notable example of rapid speciation is the European flounder (*Platichthys flesus*), which exhibits two distinct reproductive (spawning) behaviors, leading to the emergence of two separate lineages in a relatively short time frame (Momigliano et al. 2017). Localized rapid within-lineage radiation can also occur; for instance, the cyprinid fish genus *Garra* includes more than 160 species across Southeast Asia to West Africa, with six distinct ecomorphs found in a single Ethiopian river (Levin et al. 2021).

Fishes exhibit remarkable diversity not only in their physical traits but also in their diets, life-history strategies, reproductive systems, and social behaviors. Their feeding habits range from carnivory and herbivory to filter feeding, reflecting adaptations to a wide range of ecological niches. This variation extends to their life-history strategies, with some species, like the African killifish, completing their entire life cycle in just a few months (Valdesalici and Cellerino 2003), while others, such as the Greenland shark, live for up to 400 years due to their exceptionally slow metabolism (Nielsen et al. 2016). Reproductively, fishes are predominantly gonochoric, but over 450 species display functional hermaphroditism (Kuwamura et al. 2020). Modes of reproduction include oviparity, ovoviviparity, and viviparity, with a range of mating systems including polygamy and monogamy, and some species even showing parental care (Vági et al. 2024). In terms of social behavior, some species lead a solitary existence (e.g., pikes; Casselman and Lewis 1996), while others form complex social groups numbering in the thousands (e.g., schooling fishes like anchovies and herrings; Pavlov and Kasumyan 2000). Still others form smaller, more hierarchical societies or “harems” (e.g., cleaner wrasses, damselfishes; McCormick 2016; Robertson 1972; Sakai and Kohda 2001).

Finally, fishes possess exceptionally diverse sensory systems, some of which are unique to aquatic habitats (e.g. lateral line), which fundamentally shape how they perceive and interact with their surroundings. Many species have excellent vision (Douglas and Djamgoz 2012; Guthrie 1986; Pita et al. 2015), often specialized for specific light conditions in shallow or deep waters (for a review, see Marshall 2017). For example, some deepwater fishes present a high number of rod opsins that allow them to efficiently perceive both daylight and the bioluminescence of other organisms (Musilova et al. 2019). The lateral line system allows fishes to detect water movements and vibrations, and is used in both navigation and predation (Bleckmann and Zelick 2009; Gardiner and Atema 2014). Some fishes, such as sharks and weakly electric fishes, can sense electric fields (i.e., electroreception; Kalmijn 1971; Newton et al. 2019), while other species, such as catfishes, have enhanced taste buds over the entire body that are used to “smell” prey when foraging in murky waters (Atema 1971; Northcutt 2005). The adaptations of fish sensory systems to extreme habitats and the resulting diversity in sensory processing provide scientists with valuable opportunities to investigate how different sensory inputs shape cognition and behavioral strategies. Cavefishes are an interesting example of this approach, as these species have evolved in perpetual darkness and have become entirely or partially blind (Borowsky 2018; Kuball et al. 2024). By comparing cavefishes with their epigean “surface” relatives, researchers can explore how behaviors such as spatial cognition develop and function with reduced to no visual input (Kleinschmidt et al. 2024; de Perera 2004; Sovrano et al. 2018; Yoshizawa et al. 2010).

Spanning countless ecological niches and evolutionary trajectories, fishes exhibit a wide variety of traits shaped by different environmental and social pressures (Patton and Braithwaite 2015). We expect that this would also occur for cognitive traits, determining interspecific differences that may allow us to understand how selection shapes cognition. The extreme diversity of fishes, along with their ancestral relevance, allows researchers to test a wide range of evolutionary hypotheses and gain unique insights into the origins and adaptations of cognitive abilities across vertebrates. Moreover, some fishes show highly plastic cognitive abilities (Kotrschal and Taborsky 2010; Lucon-Xiccato et al. 2023; Salvanes et al. 2013), supported by variation in brain morphology (Pollen et al. 2007; Triki et al. 2022) and abundant continuous neurogenesis (Schmidt et al. 2013). This phenotypic plasticity allows them to quickly adjust to environmental demands, thus making them a valuable model for studying the relative roles of ecology and cognition in shaping behavior within species. Individual differences, which are the basis for evolutionary changes to occur, are also present in fishes and are probably the largest observed in any vertebrate (Lucon-Xiccato et al. 2020). Previous studies have already successfully simulated cognitive selective processes in the laboratory with fish populations (Kotrschal et al. 2013), establishing this taxonomic group as a promising model system for investigating the evolutionary mechanisms underlying cognition.

### Problems in fish comparative cognition

One of the main limitations of the study of fish cognition, and particularly fish *comparative* cognition, is that only a small number of species overall have been tested, and those species selected by researchers are often unusual or biased in some way. For example, experimental species are typically selected for practical reasons such as their ease of capture, local availability, survival and reproduction rates in captivity, or efficiency at learning cognitive tasks. Salena et al. (2021) highlighted that fish cognition studies predominantly involve a few overrepresented species, such as goldfish, guppy, zebrafish, and stickleback, which are all relatively small, generalist freshwater species. Moreover, 69% of the studies they analyzed relied on captive-bred fishes, which may differ significantly from their wild counterparts, and 91% were conducted within laboratory settings, limiting ecological validity (Webster and Rutz 2020). This sampling bias inevitably influences our understanding of cognition across fishes. In addition, apart from a few exceptions (e.g., Agrillo et al. 2012; Salena and Balshine 2020; Salena et al. 2022; Santacà et al. 2019a, 2020), most research is limited to single-species studies, with specific cognitive tests typically implemented by only one research group, thus limiting cross-species comparisons. Conclusions drawn from a limited number of species within *and* between studies risk oversimplifying or misrepresenting evolutionary patterns. It is therefore critical to increase the number and variety of species, functions, and settings used to comprehend cognitive evolution in fishes.

Another important limitation to the field pertains to the lack of standardized methods for cross-species comparisons. When multiple species are studied across different sites with the same research goal, inconsistencies in methodologies often arise, complicating the interpretation of results. A growing body of evidence suggests that even small modifications in experimental paradigms can lead to significant variations in cognitive assessments of fishes (Agrillo and Bisazza 2014; Gatto et al. 2017, 2021; Jones et al. 2023; Lucon-Xiccato et al. 2017a; Prétôt et al. 2016). For example, in threespine sticklebacks (*Gasterosteus aculeatus*) and European minnows (*Phoxinus phoxinus*), the size and complexity of a T-maze to assess spatial cognition influence the participation rate of fishes and significantly impact success rate, respectively (Jones et al. 2023). As another example, guppies (*Poecilia reticulata*) show astonishing numerical discrimination abilities comparable to mammals and birds, yet only if trained with 3D stimuli and not 2D stimuli, thus questioning if guppies actually apply patch quality estimation rather than numerical discrimination with 3D stimuli (Gatto et al. 2017). Even seemingly minor changes, such as altering the distance of a transparent barrier to a social reward, can influence the performance of guppies in inhibitory control tasks (Gatto et al. 2018). These findings underscore how small variations in experimental setups can yield different outcomes, potentially resulting in misleading conclusions about fish cognition.

Importantly, the above limitations highlight a central paradox in the comparative study of fish cognition: the exceptional diversity of fishes constitutes both a strength and a constraint for comparative research. On one hand, the taxonomic, ecological, and behavioral breadth of fishes provides an unparalleled opportunity to examine how cognitive processes evolve under diverse selective pressures and under environmental conditions. On the other hand, this same diversity – spanning hundreds of millions of years of evolution, multiple independent origins of key traits, and profound variation in morphology, neurobiology, and life history – renders broad generalizations about “fish cognition” inherently problematic. This is in part because fishes do not represent a monophyletic group with shared ancestry, but rather are a paraphyletic group (that excludes tetrapods), meaning that the grouping conflates lineages with deeply divergent traits. As a consequence, comparisons of cognitive performance across deeply divergent lineages using standardized assays may, in many cases, be conceptually or methodologically untenable. In fact, interpreting behavioral data without explicit phylogenetically corrected analyses and attention to ecological contextualization, may result in comparative inferences related to behavioral performances risk oversimplification, misrepresentation, or fail to capture subtle yet important differences.

Recognizing these challenges, coordinated large-scale efforts are needed to advance the study of fish comparative cognition in a systematic, integrative and interdisciplinary manner. To address issues of sampling diversity, methodological inconsistency, and limited cross-species comparability, we propose the implementation of *ManyFishes* – a big team science network dedicated to fish comparative cognition and behavior. Like other big team science initiatives, ManyFishes is grounded in open science principles that emphasize transparency, reproducibility, and inclusivity. By assembling large and taxonomically diverse samples and implementing standardized experimental protocols across laboratories, ManyFishes provides a framework to capture, rather than obscure, the cognitive diversity of fishes. This coordinated and transparent approach holds promise for overcoming the limitations imposed by the group’s extraordinary breadth, enabling more reliable and generalizable insights into the evolution of cognition.

## The ManyFishes project

### Infrastructure and goals

**Fig. 1:**
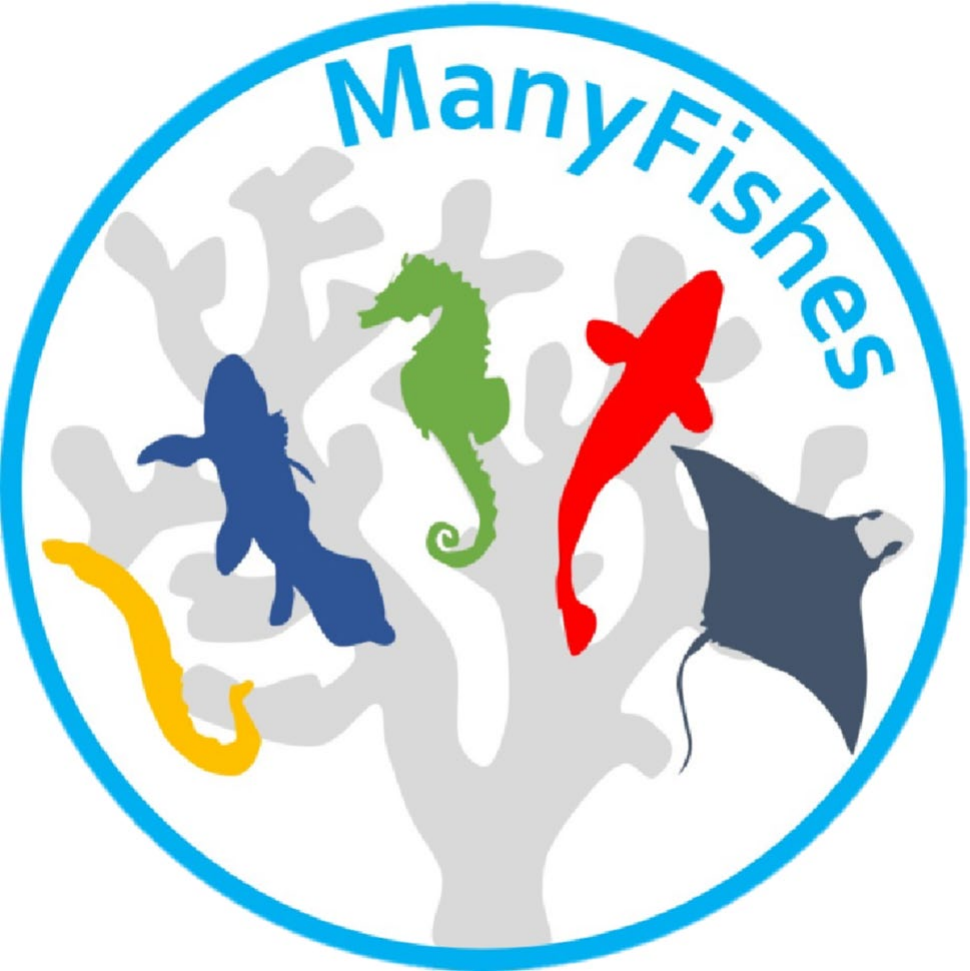
The ManyFishes project logo. The image represents the main clades of extant fishes (jawless, lobe-finned, ray-finned, and cartilaginous) and their diversity, both morphological and ecological (here represented by the seahorse and coral in the background)

**Fig. 2:**
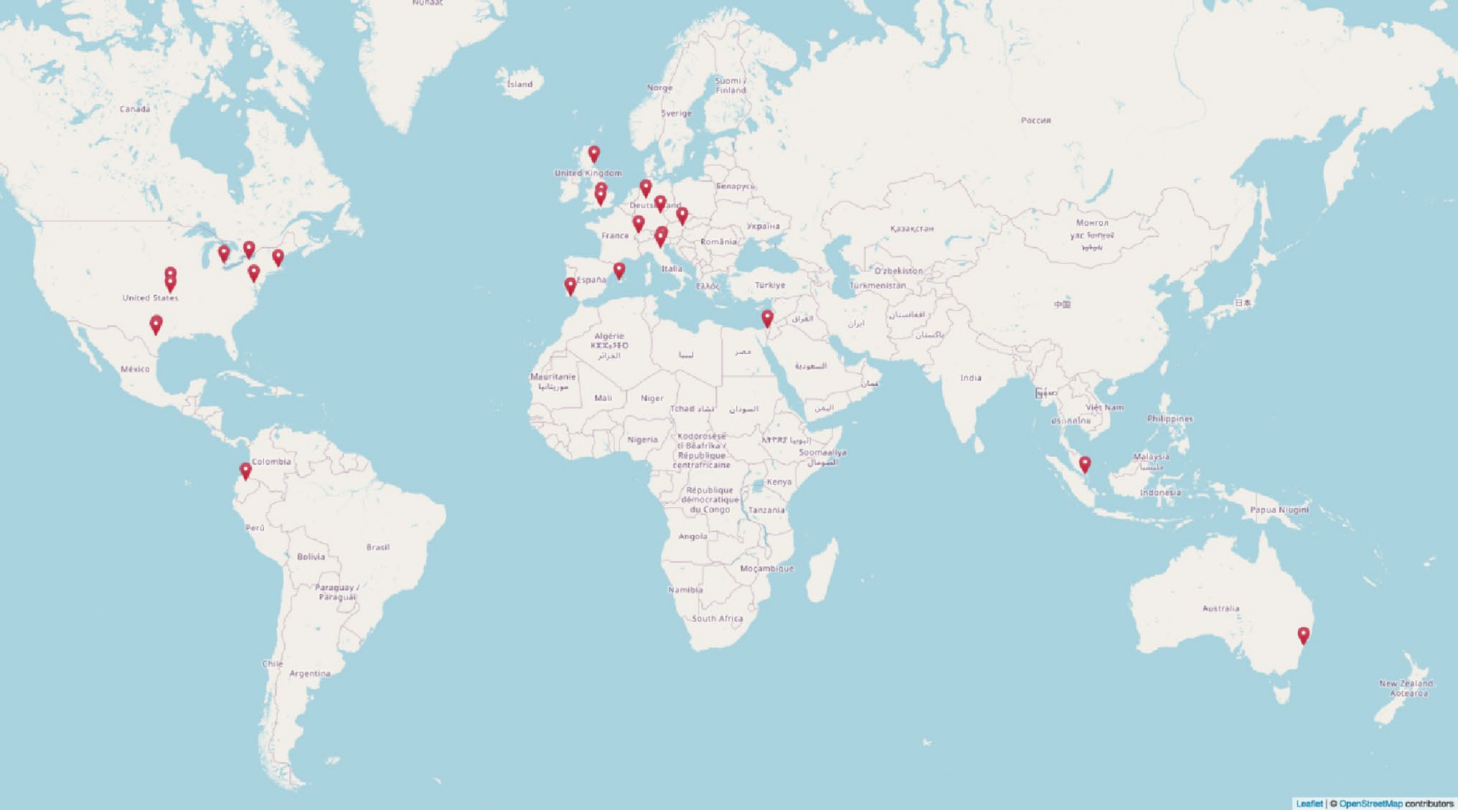
World map of the current distribution of ManyFishes collaborators across institutions. Map generated using OpenStreetMap data © OpenStreetMap contributors, created with the leaflet package in R (Cheng et al. 2025) (link: /)

ManyFishes is a large-scale collaborative project dedicated to fish cognition and behavior studies (Fig. 1). The collaboration was established in 2022 following conversations among a small group of researchers with diverse backgrounds and expertise in ichthyology. The project was officially introduced at the first Big Team Science Conference held in October 2022. Since then, the network has been holding regular meetings and has expanded its membership, now counting over 30 collaborators from more than 20 institutions and representing more than 10 countries (Fig. 2).

ManyFishes’ project infrastructure currently consists of (1) a website for general information (link: https://themanyfishes.github.io/), (2) an email address for external communication and inquiry (themanyfishes@gmail.com), (3) a mailing list for network and project updates shared with members (to join upon registration), and (4) a Slack workspace for instant messaging and group chats. The project has already developed its own code of conduct to prevent misconduct and promote equity, diversity, and inclusion goals, along with a collaboration agreement that lays out clear guidelines for communication, attribution of authorship, data sharing, and respectful collaboration. To promote accessibility and transparency across the network, ManyFishes currently communicates with collaborators using a Google Drive repository, including Google Docs (e.g., study materials, protocols, and manuscripts), Google Sheets (e.g., data entry and maintenance), Google Forms (e.g., surveys and registration), and Google Slides (e.g., presentations, FAQs). Videos illustrating procedures and pictures of stimuli and testing equipment are also accessible from the repository.

ManyFishes’ primary goal is to increase both the number and the diversity of fish samples used in comparative cognition research. To achieve this goal, ManyFishes aims to (1) facilitate collaboration across researchers and institutions, (2) develop standardized yet suitably adapted procedures for wide application across species and settings, and (3) promote the use of open science tools in fish behavior. By fostering cross-disciplinary and dynamic collaboration, ManyFishes enables the investigation of key research questions from multiple perspectives, thereby offering a more comprehensive understanding of the breadth of species’ behavior and cognition.

### ManyFishes 1

A manuscript is currently being prepared on ManyFishes’ first empirical investigation (*ManyFishes 1*), which is devoted to the study of inhibitory control. Inhibitory control is defined as the capacity to suppress automatic, impulsive, or habitual responses in favor of more appropriate ones – a fundamental component of executive functions (Diamond 1990, 2013). This capacity underlies a broad spectrum of critical decision-making behaviors, such as feeding in the presence of a predator or a higher-ranking conspecific and exploring novel environments to gain access to resources. It is also widely regarded as being closely associated with general intelligence (Beran and Hopkins 2018; Shamosh et al. 2008) and cognitive competence (Duckworth et al. 2012; Olson et al. 2002).

In fishes, for example, inhibitory control enables bluestreak cleaner wrasses (*Labroides dimidiatus*) to feed against their preference to avoid losing clients (Grutter and Bshary 2003), Nile tilapia females (*Oreochromis niloticus*) – which are mouthbrooders – to overcome the temptation to swallow their offspring (Brandão et al. 2019), and juvenile chum salmons (*Oncorhynchus keta*) and guppies (*Poecilia reticulata*) to quickly stop foraging activities under high predation pressure (Ryer and Olla 1991; Savaşçı et al. 2021). The current literature on inhibitory control seems to indicate that fishes show similarities both at the performance and mechanism levels with other vertebrate groups, such as mammals and birds (for a recent review on teleost fishes, see Lucon-Xiccato 2024).

Yet, research on inhibitory control in fishes so far is almost exclusively confined to single laboratories or groups that use small samples and single (or just a few) species, thus precluding wide-ranging comparisons and interpretations of existing evidence. The goals of ManyFishes 1 are twofold: first, build a large, species-diverse dataset to better understand the evolution of inhibitory control across fishes and, second, demonstrate the functionality and feasibility of the ManyFishes project – an approach used by other ManyX networks (e.g., Many Primates et al. 2019).

ManyFishes 1 uses a standardized version of a widely used paradigm – the cylinder task (Diamond 1990; Duque and Stevens 2022; Kabadayi et al. 2018) – to assess inhibitory control capacities across species. In the task, subjects must successfully “detour” a transparent cylinder to reach a food reward through side openings, instead of directly reaching for it and bumping into the cylinder (which is regarded as an inhibitory failure). The cylinder task is an ideal candidate for ManyFishes 1, both conceptually and methodologically. Indeed, a recent large-scale comparative study found that absolute brain volume best predicted the performance of 36 species of mammals and birds in the cylinder task, and that within primates, dietary breadth (i.e., the number of dietary categories reported to have been consumed by a species) was a correlate of cognitive skills (MacLean et al. 2014). ManyFishes 1 thus represents a unique opportunity not only to fill the gap in the inhibitory control literature of vertebrates but also to explore potential key drivers of fish cognitive evolution, including brain size and ecology. Additionally, the cylinder task is a relatively simple, minimally invasive, and versatile procedure that has been used to measure diverse cognitive skills across a wide range of animals, including fishes (e.g., Boogert et al. 2011; Bray et al. 2014; Guadagno and Triki 2024; Keagy et al. 2019; Lucon-Xiccato et al. 2017b; MacLean et al. 2014; Szabo et al. 2020; Vlamings et al. 2010), thus making it suitable for big team comparative research.

A key methodological innovation in ManyFishes 1 is the modification of the standard inhibitory control procedure to mitigate certain concerns, such as the influence of prior experience with transparent surfaces (Kabadayi et al. 2016; but see Santacà et al. 2019b). To promote greater comparability, we introduced an *acclimation* phase designed to equalize exposure to the task apparatus. During this phase, all subjects, irrespective of prior experience, were systematically introduced to each experimental component sequentially. In particular, a *cylinder familiarization* period allowed subjects to freely explore the apparatus over two days, followed by a series of *forced trials* during which they were required to enter and exit the cylinder. While this design does not fully eliminate potential effects of prior experience with transparent materials, it ensures that all individuals receive equivalent, controlled experience with the cylinder itself – providing a more standardized foundation from which to evaluate inhibitory control performance.

ManyFishes 1 has recently completed data collection and is now entering the analysis phase. The current dataset encompasses 444 individuals across 22 species of freshwater and marine teleost fishes, representing the largest sampling of ray-finned fishes to date. Being the first experimental work of the consortium, ManyFishes 1 was purposely less restrictive on species selection. Specifically, it employed an *opportunistic* approach to species selection, balancing practical considerations with the scientific objectives of the project. Importantly, priority was given to species that were readily accessible and compatible with the experimental procedures, ensuring that data collection could be conducted reliably across multiple sites and research groups. While representation across multiple species was considered, the primary aim was to establish the feasibility of conducting coordinated, large-scale studies of cognition in fishes, thus serving as a proof of concept for the broader initiative. By acknowledging the trade-offs between accessibility, methodological consistency, and the diversity of fishes, ManyFishes 1 provides a framework that can guide future efforts within the network and inform best practices for large-scale comparative research in fishes.

Upcoming analyses will examine the effect of species, natural history, age, sex, trial number, and testing site on performance. In addition, phylogenetic methods will allow reconstructing the evolutionary history of inhibitory control across fishes – an approach successfully employed by other big team science projects (e.g., Many Primates et al. 2019). Finally, the dataset will enable comparisons of fish performance in the cylinder task with other vertebrate groups, including mammals and birds (MacLean et al. 2014), helping to address a longstanding gap in our understanding of inhibitory control across vertebrates. With this first study, ManyFishes aims to inspire further collaboration, offering a platform for future large-scale studies of cognition in fishes.

### ManyFishes “N”

ManyFishes 1, as currently constructed, provides a versatile template for consistent, collaborative studies across multiple species, research groups, and institutions. Such an approach allows for a wide range of potential expansions of the original scope by altering various aspects of the methodology. So-called *spin-off* projects already exist in other big team science groups (e.g., ManyBabies). In the case of ManyFishes 1, for example, a spin-off study could involve the use of *social* rewards (rather than food rewards; e.g., access to conspecifics), which is particularly suitable when working with highly social species and/or for mitigating the effect of extraneous, motivational variables (e.g., hunger or satiety levels). More importantly, ManyFishes serves as a versatile platform for exploring a broad spectrum of fundamental research questions in the field, including how variables such as life history and sociality, shape cognition in fishes.

Life history differences in ecology, ontogeny, rearing conditions, and experience can dramatically influence subjects’ performance in behavioral or cognitive tasks. For example, the performance of bluestreak cleaner wrasses on the biological market task – a dichotomous choice task designed to simulate the cleaner-client mutualistic interaction in the wild – varies with the complexity of their natural ecology: individuals from rich environments (i.e., with high density and diversity of client species and cleaners) perform better than those from poorer environments (Triki et al. 2018, 2019; Wismer et al. 2014). Changes in environmental quality during ontogeny can also permanently affect subjects’ learning abilities later in life; for instance, cichlids who experience a change in feeding regime (from low to high or vice versa) outperform those fed constant rations in associative learning tasks (Kotrschal and Taborsky 2010).

Fishes used in assays come from a variety of sources that include wild-caught, captive-bred, and even lineages that have been reproduced in captivity to the point of domestication. Domesticated animals are likely to be subject to unique evolutionary pressures due to artificial selection, and/or an escape from multifarious selection combinations which shape the behavior of wild populations. Consequently, domesticated subjects might outperform their wild counterparts in tasks that are designed and presented in artificial settings, especially when those require training or habituating to interactions with human experimenters (Lucon-Xiccato and Bisazza 2014; Lucon-Xiccato et al. 2019; Varracchio et al. 2024). However, the opposite is also true: individuals raised in captivity and domestic strains may show inferior performance in ecologically relevant tasks relative to their wild counterparts, presumably due to a lack of experience (Bibost and Brown 2014; Gatto et al. 2018; Näslund 2021). Systematically *and* intentionally including domesticated and wild populations (or species) is an important aim in the design of ManyFishes studies. Such data can be included in statistical models to account for possible variation in the study sample. Alternatively, testing subsets of species for which access to both domesticated and wild populations is possible, can provide more robust analyses of the effect of such variables.

Fishes also vary in their levels of sociality, which might become an issue in captive settings where group size is often limited by spatial constraints (e.g., aquarium dimensions). Many species are highly social, forming shoals and schools in which individuals have evolved a dependency on conspecifics to successfully navigate the environment, avoid predation, forage, and find mates. Social fishes that naturally depend on group dynamics and social learning may thus perform differently in more isolated environments when the motivation to return to conspecifics can override the motivation to consume a food reward. For example, cichlid individuals from highly social species who are kept in isolation perform significantly worse than their group-housed counterparts in spatial tasks (Brandão et al. 2015). Similarly, rainbow trout that are socially deprived show severe learning deficits in active avoidance cognitive tasks (Ausas et al. 2019). To mitigate social isolation and the associated elevated stress, various experimental strategies exist. For example, an adjacent aquarium containing social companions can be placed near the isolated subject(s), allowing for direct visual contact with conspecifics (Agrillo et al. 2008). Combining olfactory cues with visual ones (e.g., using mesh partitions) can also help reduce anxiety during isolation (Daniel and Bhat 2022). Alternatively, mirrors may be introduced in the aquarium to create images of conspecifics (Miletto Petrazzini et al. 2012); however, it remains to determine whether these strategies are necessary for specific species and, in the case of mirrors, whether they are effective in species capable of self-recognition (e.g., Kohda et al. 2019, 2022). Inconsistencies in the application of these methods may result in variations in performance and affect cross-species investigations.

Investigating how major drivers of evolution, such as life history and social behavior, influence cognitive abilities in fishes necessitates an in-depth examination of cognitive performance across a wide range of species, ecological settings, and contexts. Such a level of inquiry can be more effectively achieved through a big team science collaborative effort, such as the ManyFishes project.

## Challenges and solutions for ManyFishes

Establishing a big team science network is inherently challenging, requiring coordination across multiple laboratories, standardization of protocols, and careful management of data collection and analysis. These difficulties become *amplified* when the focus is a phylogenetically diverse group such as fishes. Unlike largely monophyletic groups studied in other big team science initiatives (e.g., ManyBabies, ManyDogs, ManyBirds, ManyPrimates) – fishes comprise a non-monophyletic assemblage with extraordinary diversity. This diversity complicates every stage of network development, from selecting representative species and designing standardized assays, to interpreting cross-species comparisons in ways that meaningfully capture cognitive patterns without oversimplifying or conflating disparate lineages. Consequently, while big team science networks have proven effective for relatively cohesive vertebrate groups, creating a similarly coordinated framework for fishes represents an even more formidable scientific and logistical undertaking. We elaborate on these issues below.

First, selecting species for ManyFishes studies requires careful, intentional consideration. Without deliberate choices about which species to include, it can be extremely difficult – or even impossible – to draw robust inferences about the influence of ecological factors or shared evolutionary history shape patterns of cognitive and behavioral variation across fishes. The non-monophyletic nature of the group further complicates this task, as lineages differ profoundly in morphology, neural architecture, life history, and sensory systems. In the context of a collaborative initiative such as ManyFishes, it is therefore essential to provide clear guidance on species selection, balancing logistical accessibility, methodological applicability, and phylogenetic and ecological representation. Addressing this challenge helps collaborators design studies that maximize the interpretability and comparative power of the network, while acknowledging the inherent limitations imposed by the diversity of fishes.

Furthermore, developing and applying similar procedures while complying with ethical guidelines, which vary substantially between countries and affiliated institutions, can be a daunting task to accomplish. While the latter is unlikely to pose significant issues in the context of free-choice and/or passive procedures (as is the case with ManyFishes 1), research may become particularly challenging when seeking approval from ethical committees in the context of more invasive procedures, such as those employing prolonged operant conditioning techniques or requiring brain tissue analyses (e.g., DeLong et al. 2017; Gatto et al. 2021; Kotrschal et al. 2013; Triki et al. 2022). As part of its collaborative nature, ManyFishes aims to facilitate the sharing of materials and documentation used in ethical approval processes between group members, thus ensuring that protocols meet the highest ethical standards possible across the collaboration. By establishing a culture of shared knowledge and continuous improvement throughout the project lifecycle, ManyFishes becomes a valuable contributor to improving the *3Rs* principles, especially Reduction and Refinement.

Like other big team science consortia, ManyFishes works towards developing standardized cognitive tests – or batteries of tests – for application to a wide range of species. In many instances, the data collected quantify the success of a species in accomplishing the task(s). However, using the same procedure with different species can also lead to the observation that a given species is unable to accomplish a specific task because of extraneous factors (i.e., unrelated to the cognitive ability per se; Alessandroni et al. 2024a; Rowe and Healy 2014), such as experimenter bias and experimental errors, within or across testing sites (Burghardt et al. 2012; Howard and Barron 2024). To mitigate those effects, experiments should be designed collaboratively by representatives of lab/group/institution partners in order to ensure consistent methodologies, while promoting both the documentation and sharing of experimental setup, site-relevant information (e.g., apparatus proportions, aquarium dimensions, social group size; see ManyPrimates et al. 2022), and footage (e.g., walkthrough and sample videos; see ManyDogs Project et al. 2023b; The ManyBabies Consortium 2020). To better control for potential experimenter effects, big team science research can further benefit from the use of low-cost, open-source automated systems for data collection and analysis (e.g., Ajuwon et al. 2024; Dutta et al. 2025). Such measures ensure internal consistency in both methodology and interpretation of data, while facilitating transparent discussion of discrepancies to reach a consensus view among participating groups.

Another possible explanation for a species’ failure or success to show a particular cognitive ability is the lack of ecological validity of the task or assay designed to measure it. For example, species that rely on particular sensory systems (e.g., vision, olfaction) might perform better in tasks that use the same modality (Santacà et al. 2021). Even within the same sensory system, species can rely on different cues; guppies, for example, are more accurate in visually discriminating the size of food items as compared to the number of food items in a patch, presumably because of their social foraging habits (Lucon-Xiccato et al. 2015). Similarly, bluestreak cleaner wrasses outperform non-human primate species in the biological market task (Salwiczek et al. 2012; Prétôt et al. 2016). It is thus crucial to take into consideration the level of ecological relevance in the intended task when evaluating a species’ cognitive abilities and/or when selecting the task at hand (Salena et al. 2021). That being said, what might initially appear as an obstacle for cross-species comparisons can become an opportunity within the ManyFishes framework: a species’ failure to perform a task may offer meaningful insights into how ecological factors shape cognition. In this way, ManyFishes enables more accurate assessments of interspecific variation in cognitive abilities.

Besides interspecific experimental design considerations, there are also intraspecific – *individual-level* – aspects that need to be considered (Lucon-Xiccato and Bisazza 2017). Motivational factors such as hunger, reward type, and stress level can affect performance measures across individuals. For example, cichlids who solve a spatial task to gain access to females and shelter have lower cortisol levels and are more engaged in tasks than those who do not (Wood et al. 2011). Similarly, goldfish who observe a trained conspecific solve a maze are generally less motivated and more likely to accept a food reward than those without the demonstration (Blane and Holland 2024). Intraspecific variability poses a notable challenge for ManyFishes due to the project’s primary focus on interspecific behavioral variation. Precisely, such variability might complicate the choice of appropriate sample sizes: increasing replicates across sites or research groups may inadvertently amplify intraspecific variation, while small or improperly balanced samples risk insufficient statistical power or potential bias. While such issues can be partially addressed through statistical modeling, they might still necessitate careful consideration in study design. Regardless, it is important to remain open to the possibility of investigating individual and population-level differences by focusing on a limited number of species across multiple laboratories. This approach would allow for sufficiently large sample sizes to effectively analyze individual variation in traits at the individual level.

Finally, big team science groups face unique challenges associated with resource accessibility, which varies immensely across countries and institutions. Compared to the United States, Australia, the United Kingdom, and Western Europe, many nations operate with significant resource limitations (Petersen 2021). To promote inclusivity and global accessibility through open science practices, ManyFishes needs to facilitate (1) the development of protocols and methodologies that are adaptable and feasible beyond existing economical and geographical boundaries (e.g., using local resources can help limit the costs associated with the import of test equipment), (2) the provision of free training resources, such as online tutorials and workshops, to assist underserved groups with implementing their research successfully (e.g., allowing the translation of protocols in multiple languages), (3) the creation of open-access databases to enhance international collaborations and resource sharing, which can also serve as platforms for the exchange of experiences among members of the global community, and (4) the promotion/visibility of ManyFishes to as wide an audience as possible through conferences, publications, outreach, and social media. For ManyFishes, fostering inclusivity is particularly critical, as researchers in under-resourced regions may have access to understudied or rare/endangered fish species and ecosystems. Establishing such collaborations would thus enable ManyFishes to achieve a more comprehensive and globally representative understanding of fish populations and ecologies, rather than being constrained to a limited number of geographic regions.

## Scientific and social impact of ManyFishes

### Global database

Due to its large-scale, wide-reaching collaborative nature, ManyFishes can serve as a starting point for creating a large, shared and open database for fish comparative cognition. Similar databases already exist for other areas and fields within fish science, most notably *FishBase* (Froese and Pauly 2025), which covers a wide range of biological data across a broad spectrum of species, and more specific databases such as the *fair-fish database* (link: https://fair-fish-database.net/) formerly known as *FishEthoBase* – for fish welfare information (Maia et al. 2024a; Saraiva et al. 2019). These databases represent invaluable resources not only for the scientific community but also for the general public, allowing researchers to freely access and analyze large datasets, offering a global perspective of existing knowledge while also highlighting critical gaps in the literature (Humphries et al. 2023; Maia et al. 2024a, b). In other cases, researchers have formed their own databases from extensive literature reviews for specific topics, such as freshwater fish species occurrence in drainage basins (Tedesco et al. 2017). These examples highlight the usefulness of, and appetite for, such large-scale data repositories within fish science. Given the vast variety of fish species studied (and not!), the benefits of collating such data into a cohesive, centralized platform hold significant potential for advancing our understanding of fish behavior and cognition.

In the case of ManyFishes, an open database could be used for the identification of systematic and evolutionary behavioral patterns across space, time, and groups, as described in the proposed field of *macrobehavior* (Keith et al. 2023), and how other factors, such as environmental change, is affecting cognitive capacities. In addition, given the extreme diversity found in fishes, such a repository could be expanded to include neuroanatomical measurements – like sizes of brain (relative and absolute) structures and divisions (e.g., telencephalon, optic tectum, cerebellum, pallium, subpallium). A comparative neuroanatomical approach would provide critical insight into (1) the link between brain structure and function, (2) generating hypotheses about the role of specific brain regions in cognitive processes, and (3) creating computational and theoretical models of brain scaling and cognitive evolution.

A large, open, and centralized database would greatly improve access to valuable data by overcoming barriers such as paywalls and fragmented sources. Unlike data embedded in individual publications – often inconsistent in format and context – an open shared platform enables efficient discovery, integration, and comparison of datasets across species and regions. This promotes equitable access for researchers globally, especially in resource-limited settings, and supports robust meta-analyses and large-scale ecological assessments. By reducing redundancy and enhancing data reuse, such a database fosters scientific efficiency, transparency, and collaboration – crucial for tackling complex, global questions in fish science.

### Welfare and conservation

The establishment of ManyFishes enables cooperation to be used to assess, virtually, *any* area of fish science. For example, the fields of fish welfare and conservation represent critical areas in which collaborative cognitive research could make a significant impact. Fish welfare has frequently been overlooked, especially in comparison to terrestrial animals. Yet, a growing body of scientific evidence supports the view that fishes are capable of experiencing pain and fear (Braithwaite 2010; Brown 2015; Sneddon et al. 2003, 2014). By drawing parallels between the cognitive abilities of fishes and those of other better-studied vertebrates, we can gain insights into their behavioral and emotional needs. Studies on fish cognition and problem-solving abilities – including those highlighted in this review – have demonstrated that they possess a level of cognition comparable to mammals and birds (for a review, see Brown et al. 2011). This knowledge has the potential to challenge traditional views of fishes as passive and insentient beings, thereby encouraging ethically informed practices in their care and management. For example, the simple act of adding environmental enrichment to a fish aquarium has been shown to enhance the cognitive capabilities of several species (Arechavala-Lopez et al. 2020; Montalbano et al. 2022; Shen et al. 2023). Similarly, recent work indicates that cognitive enrichment techniques improve long-term welfare in zebrafish (Gatto et al. 2024; Varracchio et al. 2024).

Raising awareness of fish cognition could not only improve the ethical standards of laboratory subjects but also foster greater public concern for the conditions in which farmed fishes are kept, thereby fostering the development of more robust protective policies. Indeed, recent surveys of consumer attitudes highlight a growing demand for better welfare standards in aquaculture. For instance, a 2024 survey conducted across the European Union revealed that 91% of 9,000 participants advocated for enhanced protection of fishes, even though general awareness of aquaculture practices was found to be low (Eurogroup For Animals 2024). Furthermore, while 71% of respondents agreed that fishes are capable of feeling pain, the general public’s understanding of fish sentience and welfare remains limited. This discrepancy in awareness underscores the necessity for a more profound understanding of fish cognition, which could play a pivotal role in reshaping public perception and policy.

By deepening our understanding of fish cognition, ManyFishes’ research can bring greater attention to the ethical implications of human-fish interactions and conservation efforts. As society becomes increasingly attuned to the welfare of animals, integrating cognitive research into fish conservation strategies offers the potential to promote more ethical and sustainable practices within fish science.

## Conclusion

Over the past several decades, interest in fish cognition has grown substantially. Yet much of the existing research has been conducted in relative isolation, with individual teams working independently and relying on small, often taxonomically narrow samples. While the remarkable diversity of fishes offers a unique opportunity to examine how ecological and evolutionary pressures shape cognition, it also poses significant challenges for collecting large scale databases which permit cross-species comparison and generalization. The ManyFishes project – a grassroots big team science collaboration drawing on a large and diverse research community – was developed to address some of these challenges by promoting broader sampling diversity and implementing standardized methodologies in fish comparative research. Beyond its scientific contribution, ManyFishes functions as a vital proof of concept, shaping the future trajectory and refinement of fish comparative research more broadly. Although the initiative cannot resolve all issues inherent to studying such a diverse and non-monophyletic group, it provides a platform to foster dialogue, refine experimental designs, and coordinate efforts across laboratories. In the long term, ManyFishes aims to serve as a transformative force in the field, integrating behavioral data, ethical frameworks, and cross-species insights to advance the science of fish cognition and behavior.

## Data Availability

No datasets were generated or analysed during the current study.
